# The Role of Life History and Familiarity in Performance of Working and Non-Working Dogs (*Canis lupus familiaris*) in a Point-Following Task

**DOI:** 10.3390/ani14040573

**Published:** 2024-02-08

**Authors:** Josephine M. McCartney, David A. Leavens

**Affiliations:** 1 School of Life Sciences, University of Sussex, Falmer BN1 9QG, UK; josephinemccartney96@gmail.com; 2 School of Psychology, University of Sussex, Falmer BN1 9QH, UK

**Keywords:** object-choice task, dogs, breeds, human-animal interactions, life history

## Abstract

**Simple Summary:**

We wondered whether dogs that have been subjected to formal training to work with people were better at reading pointing cues than untrained pet dogs. We compared actively working dogs (search-and-rescue, police dogs, assistance dogs, and so on), which were all from working dog breeds (sports dogs, working dogs, herding dogs) with two other groups: (1) pet dogs not from working dog breeds (toy dogs, mixed breeds, and mongrels) and (2) pet dogs from working dog breeds. We found that both trained dogs and pet dogs from working dog breeds performed better than pet dogs from non-working dog breeds, suggesting that breeding does influence performance beyond evolutionary changes attributable to domestication, per se.

**Abstract:**

Domestic dogs are very successful at following human communicative gestures in paradigms such as the object-choice task. Pet dogs also prefer responding to cues given by a familiar cue-giver and this had not been found in working dogs. Therefore, we tested three groups of dogs in the object-choice task (*n* = 54): the groups were “Actively working” dogs from working dog breeds, pet dogs from “Non-working breeds” and pet dogs from “Working breeds”. We found that “Actively working” and “Working breeds” dog groups outperformed “Non-working breeds” in following a point in the object-choice task. We also found that both “Actively working” and “Working breeds” preferred a familiar cue-giver over an unfamiliar one, in contrast to previous findings. Therefore, we conclude that dogs’ abilities to perform well in the object-choice task is influenced by the selective history of the breed, and this is then increased by life experience and training.

## 1. Introduction

Domestic dogs are thought to have evolved from wolves around 25,000 to 40,000 years ago [1]. This makes dogs the earliest domesticated animal, predating cattle, pigs, and sheep who are thought to have been domesticated around 10,500 years ago [2]. A recent large-scale comparison of DNA in ancient and modern dogs reveals a complex history of divergence and admixture in the late Pleistocene to early Holocene; according to the authors, these complex migratory dynamics obscure a clear locus of origin for domestic dogs, but nevertheless they suggest that dogs likely evolved, originally, from a single wolf population or, less likely, multiple closely related wolf populations [1].

Dogs are currently the most common household pet, with 470 million worldwide [3], but they have also been used for working purposes since they were first domesticated, from initially helping humans with hunts to now working as search and rescue, police, herding, and assistance dogs. This means that dogs play an important social role and spend a disproportionately large amount of time around humans when compared with other species. Dogs are thought by some to have developed such human-like social skills as understanding the referential intent of deictic gestures (e.g., pointing) via the process of domestication. This was initially hypothesised by Hare and Tomasello [4], who noted the tens of thousands of years that dogs and humans have spent together, which allows substantial opportunity for co-evolutionary processes to take place. The process of domestication has been shown on a small scale by the ”The Silver Fox Experiment” (reviewed by Dugatkin [5]). Researchers selected a group of foxes (*Vulpes vulpes*) with non-aggressive traits and found that within six generations, the foxes showed signs of a domestication syndrome: including neotenous morphological changes such as floppy ears, and shorter, more rounded snouts, plus behavioural changes such as reduced levels of stress hormones [5], but see [6], for an argument rejecting the characterisation of these foxes as ”domesticated”. These foxes also outperformed non-“domesticated” foxes in following human communicative gestures [7].

The Domestication Hypothesis holds that dogs are born with the ability to understand human communication gestures. This was supported by Riedel and her colleagues [8] who found that dogs at 6, 8, and 16 weeks of age could all follow human communicative cues; because the puppies at 6 weeks were still being raised by their mothers, this suggests an innate ability provided through a history of domestication. Further support for the Domestication Hypothesis is that wolves (*Canis lupus*), despite having the same ancestors as dogs, do not show the same ability when it comes to understanding human communicative gestures (but see [9] and below). Ujfalussy and their colleagues [10] compared hand-raised and mother-raised dogs to hand-raised wolves—both groups of dogs performed better than the wolves despite having similar socialisation. Similarly, Lazzaroni and her colleagues [11] compared pet dogs, free-ranging dogs, and research dogs to hand-raised wolves (the latter two groups housed and raised in a research facility), finding that dogs approached humans more for food and a cuddle (i.e., food was not the only motivator); wolves approached only for food and were slower to do so, despite being raised in the same environment as some of the dogs tested. Lampe, Bräuer, Kaminski, and Virányi [12] compared similarly raised wolves and dogs living in packs to pet dogs and found all groups of dogs outperformed wolves on a variety of cognitive tasks. They hypothesised that pet dogs rely more on humans to make decisions as opposed to their wild counterparts, therefore, domestication has allowed them to develop an understanding of non-verbal human communicative gestures to make this easier. Dogs are willing to wait longer for rewards than wolves [13].

A common test of animals’ abilities to follow human cues is the object-choice task. In these studies, an experimenter hides a reward inside an opaque container and the subject is then presented with the container with the reward and one or more empty containers. The subject can only choose one container and will receive the reward if they choose the correct box. To indicate the correct container, a communicative gesture is used. The gesture is most commonly a type of point, however, gazing cues are also used. Anderson, Sallaberry, and Barbier [14] were the among the first to use the object-choice task with non-human primates. They found that capuchin monkeys (*Cebus capucinus*) could follow the cue only when pointing was used and failed on a gazing-only condition. Shortly later, Miklósi, Polgárdi, Topál, and Csányi [15] used the object-choice task on the first set of dog subjects; they found that dogs were able to reliably follow a point to find the rewarded container. Domestic dogs have also been found to follow human cues even when the cue is counterproductive, as some dogs chose empty cups, over cups with rewards [16]. Dogs have very powerful olfactory senses, and they will use this sense to find rewards in object-choice tasks [17]; however, when human cues are provided, dogs have been reported to privilege these cues over their own senses [18,19].

Dogs are not the only domestic animal to exhibit this trait as when tested, both dogs and cats (*Felis catus*), chose a baited container over an empty one if no cue was given but would choose the container, they saw a human approach, indicating that they are influenced by human actions [20]. However, even though dogs may show trust in humans and will initially follow counterproductive cues, they have been found to be less likely to follow a point once a human has shown to be misleading [21].

It is frequently claimed that dogs out-perform apes in paradigms such as the object-choice task, and this has been attributed to their different selective histories [22,23], but, as noted by Clark, Elsherif, and Leavens [9,24,25,26,27,28], to date, dogs and non-human primates have not been matched in testing contexts, task preparation, life history stage, or either quality or quantity of experience with humans. Therefore, as Udell and her colleagues noted [28] (p. 717), “[d]ogs’ sensitivity to human gestures may not be entirely explained by phylogenetic variables; rather, the interactions between genetic, developmental and experiential variables must be considered”.

Numerous studies have reported facilitating effects of training on dogs’ performances in a variety of object-choice tasks, demonstrating, among other things, that dogs can rapidly adapt to novel pointing postures [28,29]. McKinley and Sambrook [30] compared actively working gundogs to pet dogs and found superior performance by the highly trained dogs. Interestingly, they found, on average, gundogs of traditional gundog breeds outperformed gundogs from non-gundog breeds, this may suggest that the traditional breeds are aided by a genetic predisposition to perform better. Wobber, Hare, Koler-Matznick, Wrangham, and Tomasello [31] reported that working breeds are more skilled at using pointing gestures than non-working breeds. They also found that primitive breeds with little human contact and not bred to be pets or to perform working roles, such as the New Guinea Singing Dog, were able to follow human cues but were outperformed by pet dogs on the object-choice task. Udell and her colleagues [32] compared point-following performance on hunting, herding, and livestock-guarding dog breeds, using Airedale Terriers, Border Collies, and Anatolian Shepherds, respectively, reporting that Border Collies and Airedale Terriers performed above chance level when following a momentary distal point, and that, after training, Anatolian Shepherds were also able to perform above chance level, which is not typical of the breed. Thus, life history factors seem to matter in dogs’ performances in human-canine interactions, and we sought to examine the broad effects of training by comparing actively working dogs with dogs of both non-working breeds and working breeds (see Table 1 notes for definitions).

The types of interaction dogs have with a cue-giver is a potential factor in dogs’ performances in formal assessments. For example, dogs prefer receiving petting to vocal praise and do not distinguish between owners and strangers during these activities [33]. A similar finding was reported by Hegedüs, Bálint, Miklósi, and Pongrácz [34], who asked owners to encourage their dogs to select the correct box when an experimenter was pointing to incorrect one; they found that owners had no effect and suggested this was due to the experimenter calling the dog’s name and therefore, the experimenter and dog were engaged in communication. Thus, the effects of familiarity of interactant seem to be moderated by situational factors, such as the quality of interaction.

We were interested in whether actively working dogs were advantaged in the use of human cues by the cue-giver’s familiarity (owner/trainer vs. experimenter) and breed/working classification. We therefore compared an actively working group of dogs with pet dogs from working-dog breeds and pet dogs not from working dog breeds on an object-choice task, in which the owner/trainer or the experimenter provided cues to toy locations. We reasoned that if actively working dogs displayed an advantage over pet dogs from working dog breeds, that this would implicate a facilitatory effect of experience over breeding, and that if both of these groups of dogs outperformed pet dogs from non-working breeds, this would implicate an influence of breeding on performance. Finally, we elected to use familiar and unfamiliar toys in this study, for two reasons: (a) to avoid the potentially distracting effects of food, e.g., Ref. [35] and (b) because of the relative ease in manipulating toy familiarity, compared with food familiarity, during pandemic lockdown conditions.

We set out to answer three questions: (1) Are there systematic differences between dogs of different life histories in performance in the object-choice task? (2) Are there systematic differences between dogs of different life histories in their responses to familiar and unfamiliar cue-givers in the object-choice task? (3) Are there systematic differences between dogs of different life histories in their responses to familiar and unfamiliar toys in the object-choice task?

**Table 1:** List of breeds, age, sex, and working role for each subject.

Subj. ID	Breed	Sex	Age (M)	Working Role
Actively Working ^a^				
A1	Jack Russell Terrier X Spaniel	M	36	Search and Rescue
A2	English Springer Spaniel	F	96	Search and Rescue
A3	Scottish Terrier	F	23	Therapy dog
A4	German Shepherd	F	12	General purpose police dog
A5	Labrador	F	13	General purpose police dog
A6	Labrador	F	7	General purpose police dog
A7	Cocker Spaniel	M	84	Active drug dog
A8	Springer Spaniel	M	36	Active explosives dog
A9	Working Cocker Spaniel	M	72	Beating and agility
A10	Springer Spaniel	M	37	Victim recovery
A11	Labrador X Springer Spaniel	M	10	Assistance dog
A12	Cockapoo	F	36	Assistance dog
A13	Labrador	M	108	Assistance dog
A14	German Shepherd	F	8	General purpose police dog
A15	Working Cocker Spaniel	M	120	Agility
A16	Golden Retriever	F	120	Agility
A17	Working Cocker Spaniel	F	36	Agility
A18	Border Collie X Welsh Sheepdog	F	96	Scent work
A19	Working Cocker Spaniel	M	60	Agility
Non-working ^b^				
B1	Pug	F	24	N/A
B2	Unknown mix	M	72	N/A
B3	French Bulldog x Pug	F	36	N/A
B4	Unknown mix	F	36	N/A
B5	Maltese	F	65	N/A
B6	Unknown mix	M	12	N/A
B7	Unknown mix	F	7	N/A
B8	Cavalier King Charles Spaniel	F	120	N/A
B9	Shih Tzu X	M	8	N/A
B10	Shih Tzu X	F	72	N/A
B11	Unknown mix	M	144	N/A
B12	Shih Tzu	M	96	N/A
B13	Chihuahua	F	12	N/A
B14	Toy Spaniel X Toy Poodle	M	11	N/A
B15	Bichon Frise X Shih Tzu	F	96	N/A
B16	Unknown mix	F	96	N/A
Working breeds ^c^				
C1	Cocker Spaniel X Jack Russell	M	12	N/A
C3	Cockapoo	F	60	N/A
C4	Whippet	F	10	N/A
C5	Show Cocker Spaniel	F	36	N/A
C6	English Springer Spaniel	M	36	N/A
C7	Working Cocker Spaniel	M	48	N/A
C9	Bull Lurcher	M	16	N/A
C10	Shar-pei X Cane Corso	M	24	N/A
C11	Labradoodle	F	6	N/A
C12	Cockapoo	M	36	N/A
C13	Rottweiler X German Shepherd	M	81	N/A
C14	Cockapoo	F	96	N/A
C15	Cane Corso	F	16	N/A
C16	Dachshund	M	72	N/A
C17	Border Collie	M	11	N/A
C18	Staffordshire Bull Terrier	M	9	N/A
C19	Hungarian Vizsla	M	108	N/A
C20	Hungarian Vizsla	F	22	N/A
C21	Dachshund	F	8	N/A

Notes: ^a^ Actively Working breeds include dogs that are both actively working in the roles indicated and belong to one of the breeds identified by the American Kennel Club [36] as Working, Sporting, or Herding. ^b^ Non-working dog breeds include pet dogs that are not actively working and belonging to breeds identified by the American Kennel Club (2021) as Toy, Non-working, Miscellaneous, or in some cases Unknown. ^c^ Working breeds include pet dogs that have not been trained for a working role, but belong to breeds identified by the American Kennel Club (2021) as Working, Sporting, Herding, or Hound.

## 2. Materials and Methods

### 2.1. Participants

A total of 56 domestic dogs (*Canis familiaris*) served as subjects in this study. The subjects were divided into three groups, these were “Actively working”, “Non-working breeds”, and “Working breeds”. For the statistical analyses, 54 out of 56 dogs tested were analysed. Two dogs were excluded from the “Working breed” group (one male and one female) due to experimenter error: an unequal number of points were displayed to the familiar and unfamiliar items. This left the “Working breed” group with 19 subjects to be analysed (see Table 1 and notes for elaboration of dog categorisations).

The “Actively Working” dogs were breeds recognised by the American Kennel Club (AKC; [36]), and these were dogs which had been trained for a specific purpose. There were 19 subjects in this group, this included 9 males, 10 females with the age ranging from 7 months to 10 years (mean = 3.75 years).

“Non-working breeds” were dogs who were kept only as pets but not breeds belonging to any of the working groups. These breeds belonged to the toy dog group as defined by the AKC [36]; this group also contained (a) mixed breed dogs, where at least one of the dogs’ parents was from the toy group and the other of unknown breed, and (b) dogs of unknown breed. There were 16 subjects in this group, this included 6 males, 10 females with the age ranging from 7 months to 12 years (mean = 4.72 years).

“Working breed” dogs were also kept only as pets and included dog breeds from the herding, hound, sporting, non-sporting, terrier, and working group as defined by the AKC. This group also included mixed breeds when both breeds were from working groups. These dogs had not been trained to work a specific role. There were 19 subjects in this group which included 12 males, 9 females with an age range of 6 months to 9 years (mean = 3.10 years).

All dogs used in this study were over 6 months of age and had lived with their owners for a minimum of 2 months. For the full list of breeds, see Table 1. No pre-training was given to the dogs, and food rewards were not used. The participants were given a bag of dog treats for taking part in the experiment after the event.

### 2.2. Materials

**Figure 1:**
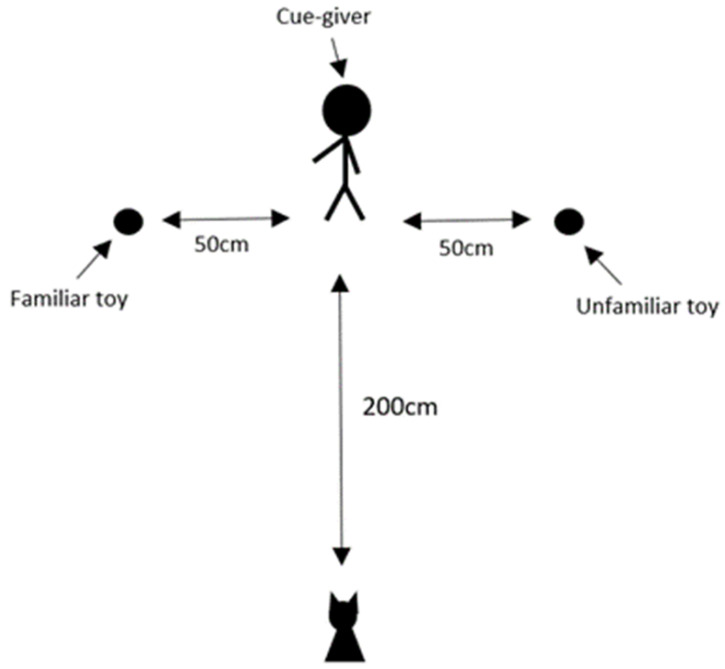
Schematic of the laboratory set up, and an example of a point towards a familiar item. Drawing not to scale, distances are approximate.

The dogs were tested in Pevensey Dog Laboratory (2.5 m in length and 4.55 m in width), to ensure standardisation in the testing context (see Figure 1). The subjects were filmed on a Sony HDR-PJ410 Handycam video camera that was set up prior to the subjects entering the room. A questionnaire was used to assess the subjects’ social experience, age, lifestyle habits, and training level to see if this affected the results (see Appendix A). This involved questions such as: On a scale of 1 to 10 how well does your dog follow commands?

### 2.3. Design

A repeated measures design was used, with two independent variables: the familiarity of the cue giver (familiar, unfamiliar) and the familiarity of a toy (familiar, unfamiliar). The first author served as the unfamiliar cue-giver and the dog owner served as the familiar cue-giver. We asked the owners to bring in a familiar item for the dogs (i.e., favourite tennis balls/toys). The unfamiliar items were a novel stuffed toy or tennis ball. The dependent variable was whether the subject (a) chose the correct referent, (b) chose the incorrect referent, or (c) made no choice. Each subject took part in all conditions of this study.

### 2.4. Procedure

**Figure 2:**
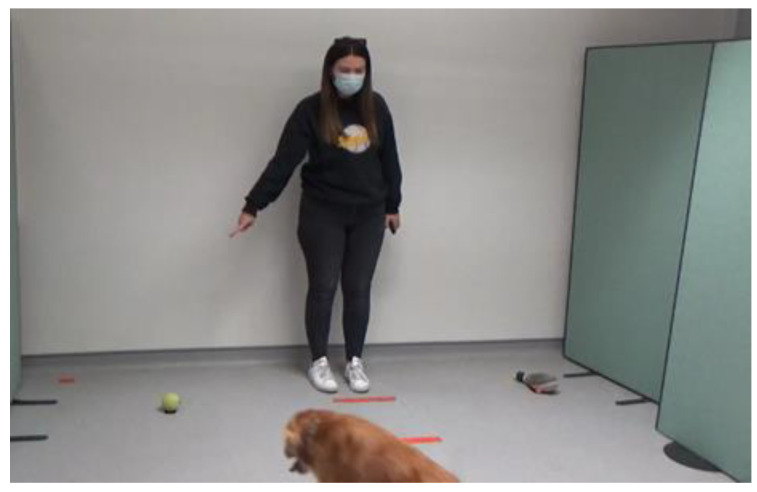
Example of trial with an unfamiliar cue-giver to unfamiliar toy.

**Figure 3:**
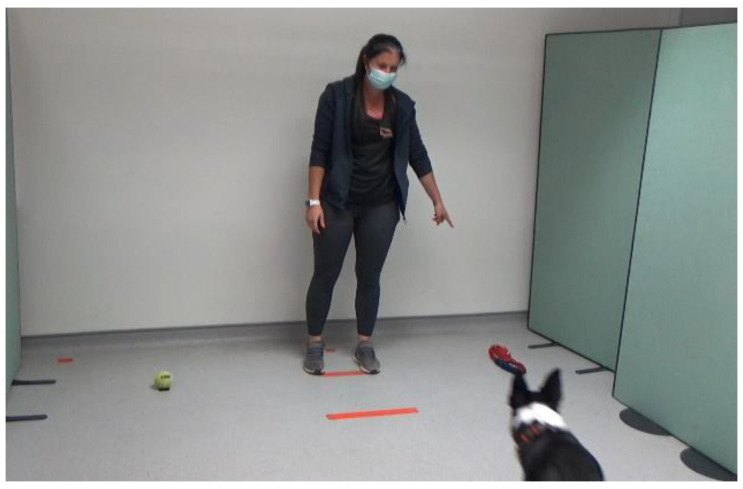
Example of trial with a familiar cue-giver to familiar toy.

The dogs were allowed off lead in the laboratory, with the owner and experimenter present to allow the dog to feel comfortable in the room. To ensure the dogs were not under stress, they were required to willingly approach the experimenter and eat from their hand. If the dogs apparently did not want to do this, then they would not take part in the experiment (following the protocol in Ref. [37]). However, no subjects were excluded on this basis. Once the dog met the inclusion criteria, the study began. Both the owner and the experimenter served as cue-givers, in counterbalanced order across the dogs. The cue-giver (either owner or experimenter) guided the dog to the starting position, which was marked on the floor of the laboratory. The human not acting as the cue-giver stood silently in a central position behind the dog and held the dog in place until the cue-giver arrived in the correct position. Commands such as ”sit” and ”stay” were used to keep the dog in the starting position. Once the dog and cue-giver were in their starting positions, ostensive cues such as calling the dogs’ name to attract their attention were used. The cue-giver pointed to one of two items (see Figure 1, Figure 2 and Figure 3); this was either an item that was familiar to the dog, or an item that was unfamiliar.

Each dog received eight trials, four with the owner as the cue giver and four with the experimenter as the cue-giver, so that each cue-giver pointed to each object once. The order in which the cues were given (left or right) were counterbalanced to reduce a response pattern bias forming, as well as the order in which the familiar and non-familiar cue giver gave the cue. All subjects received the same type of cue, the cue-giver used an ipsilateral static point, whereby the pointing hand on the same side as the intended target was in place before the participant viewed the cue and remained so until the participant chose one of the items. The cue giver kept their head directed centrally, and body position facing forward, and the cue-giver looked at the dog whilst giving the cue, to indicate the cue is meant for the dog.

If the dog did not make a choice or approached one of the two items after 1 min this was classed as a no-choice trial (as seen in similar protocols: [29,37]). A successful trial was when the dog chose the correct referent by touching it, retrieving it, or coming within 10 cm of it. An unsuccessful trial was when the subjects approached the incorrect item. During the experiment, our plan was to exclude a dog if they exhibited urination, defecation, destructive behaviour, or excessive vocalisation (these are signs of anxiety), however, no dog was excluded on these grounds.

### 2.5. Spontaneous Variations on the Form of the Pointing Cue

During the initial coding, we noticed that some owners/trainers did not point with their index fingers, as had been displayed by the experimenter, and this non-canonical pointing seemed to be more frequent in the trained working dog group; therefore we added a code for conformity with an index-finger point to determine the distribution of these points among the groups.

### 2.6. Statistical Analyses

Tests of normality showed the performance data were not normally distributed and so we elected to test our statistical hypotheses with nonparametric statistics. Kruskal–Wallis *H* tests were used to compare the three groups on the dependent variable and Mann–Whitney *U* tests were used for the pairwise comparisons between groups. Spearman’s *rho* was used to assess the relationship between overall performance and the results of the survey, and Cochran *Q* tests were used to measure the frequency of no-choice trials, and Wilcoxon signed ranks tests were used to compare two related samples.

### 2.7. Interobserver Reliability

All eight trials from eight subjects were randomly selected from the corpus and independently coded for performance by a naïve coder, who was blind to the hypotheses under test; this represents ~15% of the corpus. Reliability for dogs’ performances was Cohen’s *kappa* = 0.74, indicating excellent inter-observer reliability.

### 2.8. Ethical Note

All subjects were volunteered by their owners and were recruited through posts on social media. The dogs remained with their owners throughout this study, and the owners gave written consent for their dog to partake and to be filmed prior to this study; they were also reminded that should they wish to they could withdraw their participation at any time and leave. This study was conducted under ethical approval from the Animal Welfare and Ethical Review Body (AWERB) of the University of Sussex.

### 2.9. Risk Assessment

This study took place during the COVID-19 pandemic, therefore we needed to ensure the safety of the participants during testing. All participants wore a mask and remained a social distance of two metres when possible. The laboratory was sanitised between each participant and spare gloves, masks, and antibacterial hand gel were available. The experimenter was tested regularly for COVID-19 and all participants were invited to watch a video detailing the university’s COVID procedure.

## 3. Results

### 3.1. Sex and Age Distributions

A chi-squared analysis showed that sex ratio was not significantly different across the three groups, *X*^2^ (2, *n* = 54) = 1.45, *p* = 0.48. A Kruskal–Wallis test showed that the three groups of dogs were not significantly different in median age (*H* = 2.04, *df* = 2, *p* = 0.36). The median ages for the three groups were 36.0 months (IQR = 83) for the “Actively working”, 50.5 months (IQR = 84) for “Non-working dogs”, and 24.0 months (IQR = 49) for the “Working breeds”. These results show us that there were no obvious confounds with sex or age between the three groups.

### 3.2. Order and Side Effects

We did not find an effect of cue-giver order on overall performance, (Mann–Whitney *U* = 304.50, *df* = 1 *p* = 0.30); thus, we found no evidence that whether the experimenter or owner first served as cue-giver had any influence on results. There were six potential side orders in which the cue-givers could point, and we found no effect of the order of side of pointing (Kruskal–Wallis *H* = 5.09, *df* = 5, *p* = 0.41); this means that we found no evidence of side biases or trial order effects on performance. There were also six potential target orders in which the cue-givers could point, we found no effect of the order to which the familiar and non-familiar toys were pointed at (Kruskal–Wallis *H* = 2.45, *df* = 5, *p* = 0.78; see Table 2 for full list of order possibilities). These results show us that the trial order and the side pointed to did not seem to influence overall performance.

### 3.3. Age, Sex, and Neuter Status

All three groups of dogs were just as likely to be neutered, *X*^2^ (2, *n* = 54) = 2.50, *p* = 0.29. We found no relationship between age and performance (Spearman’s *r*_s_ = 0.05, *n* = 54, *p* = 0.71). In addition, we found no relationship between dogs’ sex and performance (Mann–Whitney *U* = 342.50, *df* = 2, *p* = 0.70). Therefore, we do not further consider these variables.

### 3.4. Performance across Groups

**Figure 4:**
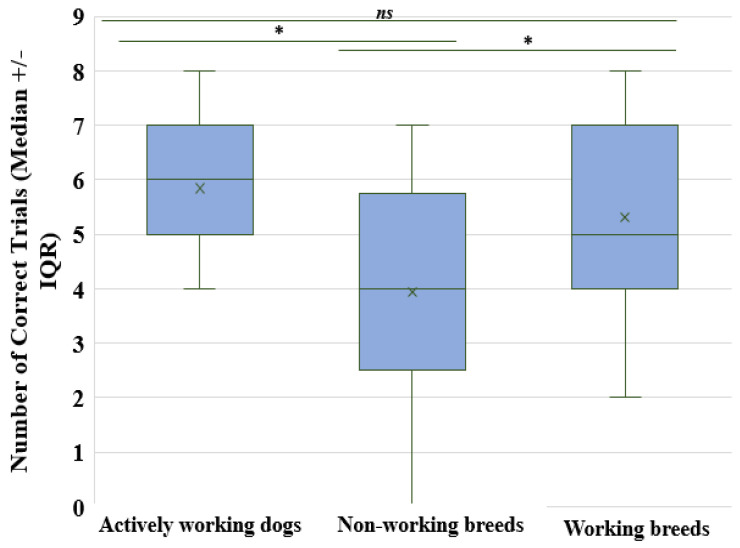
A boxplot showing the median performances and interquartile ranges (IQR) for each group. * = *p* < 0.05. *ns* = not significant.

There was a significant difference in overall performance between the three groups of dogs (Kruskal–Wallis *H* = 8.90, *df* = 2, *p* < 0.01). To look at this in closer detail for each group, we found that “Actively Working” dogs performed better than “Non-working breeds” (Mann–Whitney *U* = 64.50, *df* = 2, *p* < 0.001). “Working breeds” performed better than “Non-working breeds” (Mann–Whitney *U* = 94.50, *df* = 2, *p* = 0.05), and we found no significant difference in performance between “Actively working” dogs and “Working breeds” (Mann–Whitney *U* = 150.00, *df* = 2, *p* = 0.36). Figure 4 shows the overall performance for each group. These results show us that “Actively working” and “Working breeds” performed better overall than “Non-working breeds”.

### 3.5. Survey Results

**Figure 5:**
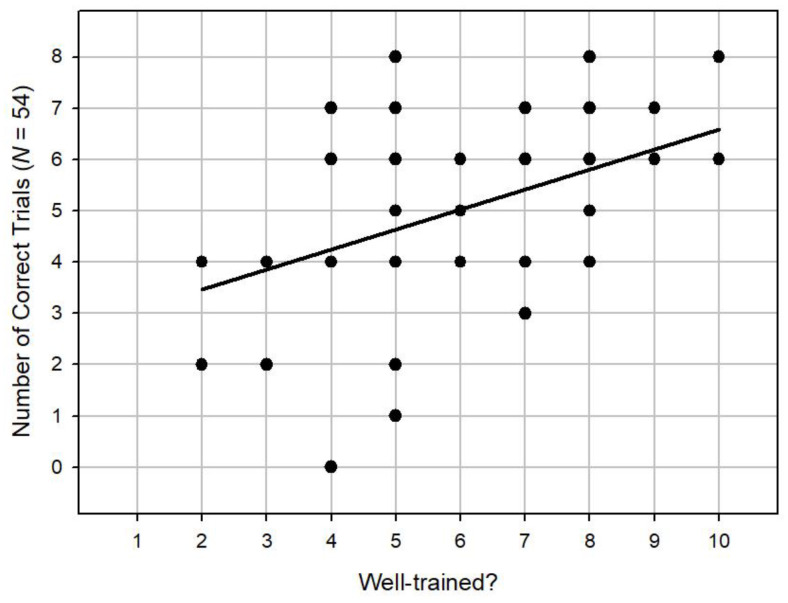
A scatterplot showing a positive correlation between the degree to which owners say their dogs are well trained and overall performance.

**Figure 6:**
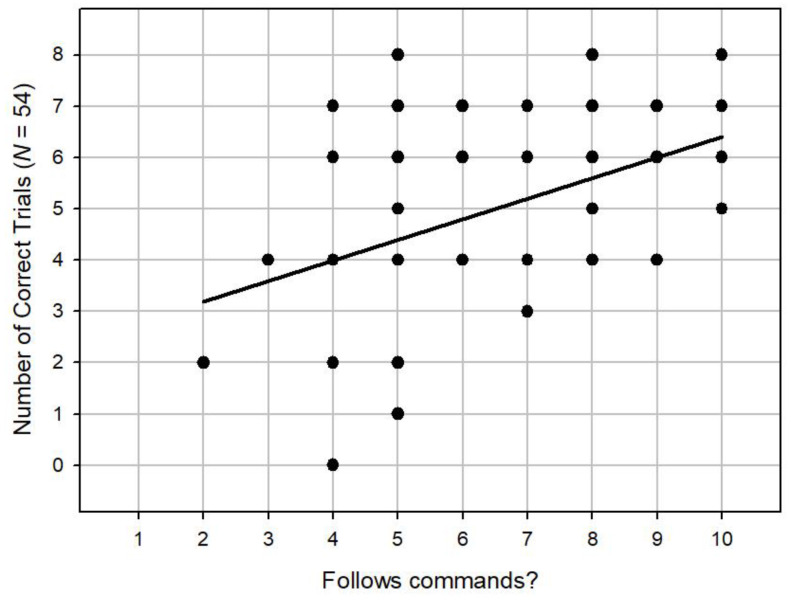
A scatterplot showing a positive correlation between the degree to which owners say their dogs follow commands and overall performance.

**Figure 7:**
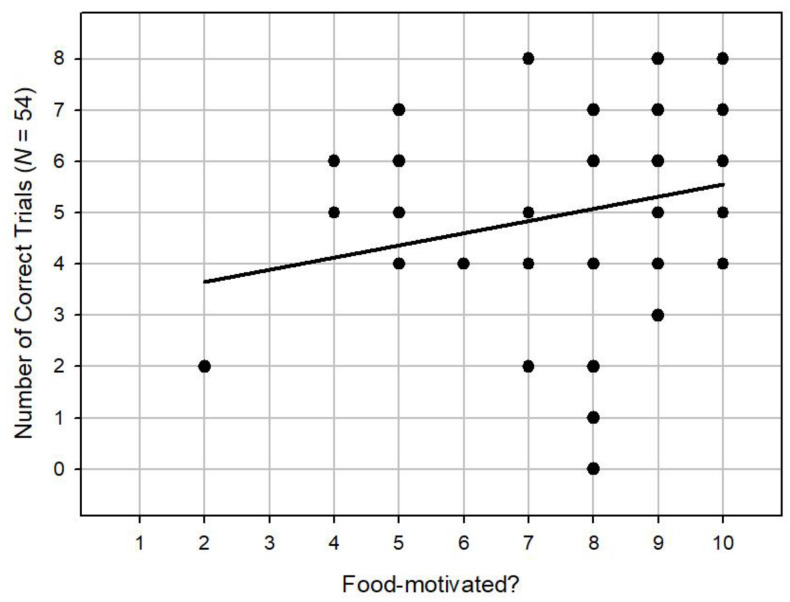
A scatterplot showing a positive correlation between the degree to which owners say their dogs are food motivated and overall performance.

We then moved on to analyse the survey questions which were given to the owners prior to the experiment. We found no correlation between the degree to which the owners say their dogs get on with other dogs and overall performance (Spearman’s *r_s_* = -0.10, *n* = 54, *p* = 0.46). There was no significant correlation between the degree to which owners say their dogs play with toys and overall performance (Spearman’s *r_s_* = 0.14, *n* = 54, *p* = 0.31). We did, however, find a significant positive correlation between the degree to which owners say their dogs are well trained with overall performance (Spearman’s *r_s_* = 0.38, *n* = 54, *p* < 0.001), as shown in Figure 5. In addition, we found a significant positive correlation between the degree to which the dogs follow commands and overall performance (Spearman’s *r_s_* = 0.39, *n* = 54, *p* < 0.001), as shown in Figure 6. Finally, we found a significant positive correlation between the degree to which owners say their dogs are food motivated between and the degree to which the dogs follow commands and overall performance (Spearman’s *r*_s_ = 0.30, *n* = 54, *p* < 0.05), as shown in Figure 7.

### 3.6. Familiarity of Cue-Giver

**Figure 8:**
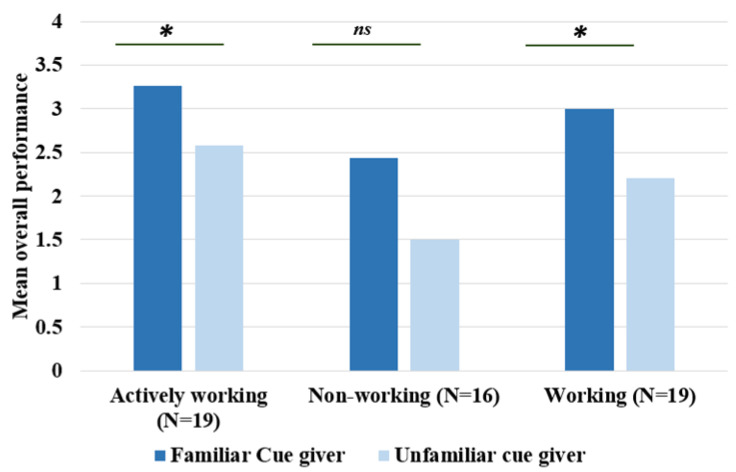
A bar chart showing mean performance for “Actively working”, “Working breeds”, and “Non-working breeds” with familiar and unfamiliar cue-givers. * = *p* < 0.05. *ns* = not significant.

We found that the familiarity of the cue-giver had a significant effect on overall performance (Wilcoxon *Z* = −3.35, *p* < 0.001). When we looked at this between groups, we found that familiarity of the cue giver had a significant effect on performance for “Actively working” dogs (Wilcoxon *Z* = −1.97, *df* = 1, *p* < 0.05) and “Working breeds” (*Z =* −2.02, *df* = 1, *p* < 0.05) but not “Non-working breeds” (*Z* = −1.81, *df* = 1, *p* = 0.07). This means that both “Actively working” and “Working breeds” responded significantly better to a familiar cue-giver than an unfamiliar one, this is shown in Figure 8.

### 3.7. Familiarity of Toy

We found that the familiarity of the toy did not have a significant effect on overall performance (Wilcoxon *Z* = −0.73, *df* = 1 *p* = 0.46). Familiarity of the toy also had no significant effect on overall performance for “Actively working” dogs (Wilcoxon *Z* = −0.48, *df* = 1, *p* = 0.63), “Working breeds” (Wilcoxon *Z* = −1.10, *df* = 1, *p* = 0.27) and “Non-working breeds” (Wilcoxon *Z* = −0.73, *df* = 1, *p* = 0.46). Thus, we found no evidence of any influence of the familiarity of the toy on performance for any group.

### 3.8. No-Choice Trials

**Figure 9:**
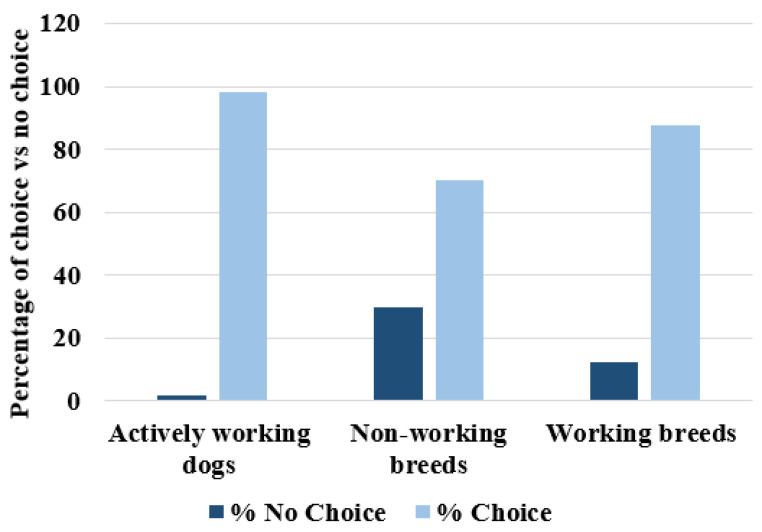
A histogram showing the percentages of making a choice vs. not making a choice for “Actively working”, “Non-working breeds”, and “Working breeds”.

We categorised each of the eight trials for every dog as either correct choice or not, and then we looked to see whether there was any systematic change in this measure over the course of the eight trials. We found that overall performance did not change over time (Cochran’s Q (7) = 6.85, *p* = 0.45). However, when we categorised each of the eight trials for every dog as either a choice was made (whether correct or not) or no choice was made, we found that the groups differed significantly in the number of no-choice trials (Kruskal–Wallis *H* = 13.75, *df* = 2, *p* < 0.001). To look at this in more detail we compared “Actively working” dogs to “Non-working breeds” and found that “Non-working breeds” had significantly more no choice responses (Mann–Whitney *U* = 57.00, *df* = 2, *p* < 0.001). We then compared “Non-working breeds” to “Working breeds” and again found that “Non-working breeds” had significantly more no choice responses Mann–Whitney *U* = 92.50, *df* = 2, *p* < 0.05). We found no significant difference between “Actively working” and “Working breeds” (Mann–Whitney *U* = 147.50, *df* = 2, *p* = 0.34). This is shown in Figure 9.

We then looked at whether no-choice trials were changing over time for each group, we did this by categorising each of the eight trials for every dog in each group as either a choice was made (whether correct or not) or no choice was made, and then we looked to see whether there was any systematic change in this measure over the course of the eight trials. We found no significant effect for “Actively working” dogs (Cochran’s Q (7) = 11.42, *p* = 0.12). This means the number of no-choice trials did not increase over time for this group, as there were only 3 no-choice trials out of a possible 152. We found no significant effect for “Non-working breeds” (Cochran’s Q (7) = 12.42, *p* = 0.08). This means the number of no-choice trials also did not increase over time; however, this group had the highest number of no choice responses with 38 out of a possible 152. Finally, we found no significant effect for “Working breeds” (Cochran’s Q (7) = 6.21, *p* = 0.52). This means the number of no-choice trials did not significantly increase over time, and this group had 19 no-choice trials out of a possible 152. These results show that the number of no-choice trials remained broadly steady throughout this study for each group.

## 4. Discussion

Our results show that actively working dogs and working breeds perform better than non-working breeds on the object-choice task. These findings are consistent with those of McKinley and Sambrook [30] and Wobber and her colleagues [31], who found that actively working dogs perform better than pet dogs. These findings are also consistent with those of Udell and her colleagues [32], who found that traditional working breeds perform better in the object-choice task. Similarly to McKinley and Sambrook [30], we compared working breed dogs kept as pets to non-working breed dogs kept as pets and found that working breeds perform better; however, unlike McKinley and Sambrook, we did not find a statistically significant difference between actively working dogs and working breeds kept as pets. Therefore, our findings are consistent with the idea of a breeding-related advantage for working dogs, although it should be acknowledged that we did not equate the pet working dogs and the pet dogs from working dog breeds on the quality or quantity of their pre-experimental interactions with their owners. Because there was no statistical difference in performance between the “Actively working” and “Working breed” groups, and both of these groups outperformed the pet dogs from non-working dog breeds, we conclude that breeding may play a role in performance in this task environment, given our sampling sizes and sampling environment. However, as emphasised by an anonymous reviewer, social experiences with humans are also clearly relevant to performance in understanding human communicative cues (e.g., [28,37,38]).

We also found that the familiarity of the cue giver has a significant effect on overall performance, such that actively working and working breed dogs respond better to cues given by their owners than by a stranger. However, no such effect was found in non-working breeds kept as pets. These findings are only partially congruent with those of Cunningham and Ramos [39], who reported that all the dogs they tested preferred their owners over a stranger; however, the breed sampling in their study differed from ours, as all dogs in their pet and highly trained categories were of working breeds, with no non-working breeds used.

The present results also indicate that previous studies conducted on dogs without their owners present may not have reflected the dogs’ true abilities to follow human cues, as suggested by Burani, Barnard, Wells, Pelosi, and Valsecchi [40]. Our results also support the findings of previous studies that have found that dogs largely react better to their owners than a stranger [17,41]. However, our results are not consistent with those of Scandurra et al. [42] and Lazarowski et al. [43], who found that actively working dogs responded just as well to a stranger as they did to their owners. In fact, we found the opposite results: in our study, non-working breed dogs kept as pets responded to strangers and owners equally. The difference in findings in this case may lie in the types of actively working dogs tested. Scandurra et al. [42] exclusively used water rescue dogs and Lazarowski and her colleagues [43] only used detection dogs in their studies; these are both types of jobs which require the dog to act on their own and not rely on a human to guide them; whereas, in our study, we used a wide range of dogs which included detection and search and rescue dogs, but also included assistance and therapy dogs who rely much more on owner interaction for their working role. We also wanted to find out whether the familiarity of the object being pointed to would affect the performance of the dogs. However, we found no significant effect of referent familiarity for all three groups on overall performance.

We then moved on to analyse the results of the survey given to the owners prior to the experiment. We found no significant correlation between the degree to which owners said their dogs get on with other dogs or the frequency the dogs play with toys and overall performance. However, we did find significant positive correlations between the degree to which owners say their dogs are well-trained and performance in this object-choice task, and we found significant positive correlations between the degree to which owners say their dogs follow commands and performance in this object-choice task. This suggests that dogs who are perceived by their owners to be well-trained do perform better on the object-choice task as these dogs may have received more training and could be perceiving the point as a command to be followed. Interestingly, we found a positive correlation between the degree to which dogs were food motivated and overall performance; because we did not use food rewards in this study, this was a surprising result. However, because most dogs are trained with food rewards, those who are more food motivated may have expected a treat once they followed the point.

The survey was measured on a 10-point scale for most of the questions and an ordinal scale for others (Yes/Sometimes/Usually/No). Because the owners were answering the survey to characterise their dogs, it could have been subjected to a variety of response biases. For example, the question “How well trained would you say your dogs is?” could be interpreted in a number of different ways pertaining to the dog’s general demeanor. Still, several items did positively correlate with the dogs’ actual performance, suggesting that owners do have a fairly good grasp of their dogs’ performance potential.

We then examined the no-choice trials in terms of frequency and changes over time. We compared the overall performance for each group and found they did differ significantly, by group classification, in the number of no-choice trials. We found that “Non-working breeds” had significantly more no-choice trials than “Actively working” and “Working breeds”, and that “Actively working” and “Working breeds” did not differ from each other significantly in the number of no-choice trials. This shows that “Actively working” and “Working breeds” are more motivated to participate in the point-following task. When we look at the exact number of no-choice trials, we can see that “Actively working” dogs nearly always made a choice, with only three no-choice trials out of 152 total trials. Therefore, we can conclude again that “Actively working” and “Working breeds” may benefit from a genetic predisposition to not only perform better in the object-choice task but to be more motivated than non-working breeds.

We wanted to see if no-choice trials were increasing over time, to indicate whether the dogs were either losing interest over time (i.e., if these no-choice trials were increasing) or, alternatively, if the dogs were learning what to do (i.e., if the no-choice trials decreased over time). However, we found no significant effect of the passage of time; all three groups remained motivated at seemingly constant levels throughout the experiment.

This study has a number of limitations. First, this study took place during the COVID-19 pandemic, which means that all participants and the experimenter were required to wear masks for the duration on the study. Because the masks cover the lower half of the face this may have been confusing to the dogs who are not used to their owners interacting with them in this way. However, because this study took place in the United Kingdom, in May and June of 2021, it seems likely that the dogs will have had some interaction with people wearing masks, previously. However, it would be interesting to complete this study again with no masks used, when it is safe to do so, to see if the same effects are found. Second, we are grateful to an anonymous reviewer for pointing out that dogs of non-working breeds tend to be smaller than working dog breeds, and therefore our specific findings of poorer performance by non-working dog breeds is potentially confounded with body size, which we did not measure—the reviewer pointed us towards McGreevy et al. [44] for further information about the effects of size on performance in dogs. Third, we thank an anonymous reviewer for pointing out that dogs’ individual preferences for the toys were not assessed—theoretically, this means that, at the group level, rather than failing to demonstrate that dogs displayed no apparent bias for or against the toys on the basis of their familiarity, it could be the case that relatively equal numbers of dogs displayed preferences for the familiar and unfamiliar toys, respectively, with results cancelled out at the group level. Finally, our sample size is relatively modest, which suggests that one possible explanation for some of our findings that diverge from other similar studies might be attributable to sampling error.

## 5. Conclusions and Future Research

Therefore, based on the findings of our study, we conclude that breed differences may play a role in dogs’ responses to communicative cues. The findings of this study do not align completely with that of the Domestication Hypothesis, insofar as a strong version of that model predicts no breed differences. Our findings suggest that a dog’s ability to perform well in the object-choice task may be influenced more by the genetics of the breed than the genetics of the species, and that this is then likely amplified by life experience and training, as suggested by Sommese, Nováková, Šebková, and Bartoš [45]) in a different experimental context (impossible task), and, in object-choice tasks by many others (e.g., [28,37,42]).

In future, these findings could be built on further with longitudinal designs, by testing non-working breeds without any training, and then training them specifically on the task to see if performance improves. This could possibly be used to see if non-working breeds could be trained for working roles. It would also be interesting to use shelter dogs, of working and non-working breeds to see whether having a stable home environment affects a dog’s ability to be trained for working roles, because, currently, working roles are performed by mainly pedigree breed dogs who are bred specifically for their individual role. If studies can find that non-working breed dogs can be trained to perform these roles just as well, it could possibly alleviate the number of dogs in shelters without homes.

## Figures and Tables

**Table 2:** List of all possibilities for cue-giver, side of baiting, and point target order.

Cue-Giver Order	Side Order	Target Order
Experimenter	LLRR	FFUU
Owner	LRLR	FUFU
	LRRL	FUUF
	RRLL	UUFF
	RLRL	UFUF
	RLLR	UFFU

Note. F = Familiar item, U = Unfamiliar item, L = Left, R = Right.

## Data Availability

Data contained within this article.

## Appendix A

Survey
Owner name:
Dog name:
Breed:
Age:
Sex:
Neutered? (Y/N)
Working dog? If yes, as what?
Time spent working (Years):
Adopted? If yes, at what age?
Time spent at shelter (if known) in years:
How well trained would you say your dogs is? Scale 1 (not at all)–10 (extremely well)
How well does your dog follow commands? Scale 1 (not at all)–10 (extremely well)
Is your dog highly motivated by food? Scale 1 (not at all)–10 (extremely)
Does your dog get along with other dogs? Yes/Usually/Sometimes/No
Does your dog play with toys regularly? Yes/Usually/Sometimes/No
